# Influence of Nano-SiO_2_ Content on Cement Paste and the Interfacial Transition Zone

**DOI:** 10.3390/ma16186310

**Published:** 2023-09-20

**Authors:** Shaofeng Zhang, Ronggui Liu, Chunhua Lu, Junqing Hong, Chunhong Chen, Jiajing Xu

**Affiliations:** 1 School of Transportation and Civil Engineering, Nantong University, Nantong 226019, China; zsfgh@ntu.edu.cn (S.Z.); hongjq@ntu.edu.cn (J.H.); 2 Faculty of Civil Engineering and Mechanics, Jiangsu University, Zhenjiang 212013, China; liurg@ujs.edu.cn (R.L.); lch79@ujs.edu.cn (C.L.); chench@cczu.edu.cn (C.C.); 3 Nantong Key Laboratory of Intelligent Civil Engineering and Digital Construction, Nantong University, Nantong 226019, China; 4 School of Urban Construction, Changzhou University, Changzhou 213164, China

**Keywords:** nano-SiO_2_ content, interfacial transition zone, digital image correlation, elastic modulus, ITZ thickness

## Abstract

Nano-SiO_2_ (NS) is widely used in cement-based materials due to its excellent physical properties. To study the influence of NS content on a cement paste and the interfacial transition zone (ITZ), cement paste samples containing nano content ranging from 0 to 2% (by weight of cement) were prepared, and digital image correlation (DIC) technology was applied to test the mechanical properties. Finally, the optimal NS content was obtained with statistical analysis. The mini-slump cone test showed that, with the help of superplasticizer and ultrasonic treatment, the flowability decreased continuously, as the NS content increased. The DIC experimental results showed that NS could effectively improve the mechanical properties of the cement paste and the ITZ. Specifically, at the content level of 1%, the elastic modulus of cement paste and ITZ was 20.95 GPa and 3.20 GPa, respectively. When compared to that without nanomaterials, the increased amplitude was 73.50% and 90.50%, respectively. However, with the further increase in NS content, the mechanical properties decreased, which was mainly caused by the agglomeration of nanomaterials. Additionally, the NS content did not exhibit a significant effect on the thickness of the ITZ, and its value was maintained at 76.91–91.38 μm. SEM confirmed that NS would enhance the microstructure of both cement paste and ITZ.

## 1. Introduction

Cement as an important cementitious material has been widely used in engineering–construction industry for a long time. This is mainly attributed to its advantages of low cost, easy availability, good mechanical properties, etc. [1,2]. However, the properties of concrete structures, especially the bearing capacity and durability in various corrosive environments, have become a major concern [3]. For example, the rapid invasion of aggressive ions in the marine environment can permeate into concrete, causing premature corrosion of steel bars in a concrete structure and reducing its service life [4,5].

Therefore, researchers have attempted to add different kinds of nanomaterials to cement-based materials to improve their mechanical properties and durability [6,7]. Wang et al. [8] proved that nanomaterials can improve various properties of cement and concrete. Alireza et al. [9] found that the rheological characteristics reduced as increase in nanoparticles and micropores in ITZ were reduced with the addition of nano-Al_2_O_3_. Chen et al. [10] presented that nano-TiO_2_ was able to provide sufficient nucleation sites to promote hydration and fill the porous structure, despite an absence of the pozzolanic effect. Gao et al. [11] revealed that carbon nanotubes not only generated higher hydration reaction, but also created net-form distributions in the hardened concrete to reinforce the ITZ. Zhu et al. [12] reported that carbon nanofiber significantly improved the elastic modulus of the ITZ, thus enhancing the Young’s modulus of concrete.

When compared to the above nanomaterials, NS has the greatest advantage in the potential pozzolanic reaction with cement hydration products [3,13]. Quercia et al. [14] demonstrated that NS efficiently used in self-compacting concrete can improve mechanical properties and durability. Zhang et al. [15] reported that when 3% of NS was added in concrete, the compressive and flexural strength increased by 15.5% and 27.3%, respectively. Aleem et al. [16] concluded that 5% NS could lead to the formation of homogeneous, dense, and compact microstructure and improve the 28 d compressive strength by 60%. Additionally, Gong et al. [17] found that when the NS content was 4%, the 28 days compressive strength of foam concrete was increased by 12%, and when NS content was 15%, the compressive strength was only amplified by 9.1%. Wu et al. [18] concluded that excess addition of nano-SiO_2_ and nano-CaCO_3_ could result in a reduction in mechanical properties, due to difficulties in ensuring sufficient dispersion. Above all, it is very effective to use NS to improve the performance of cement-based materials, but it should be mentioned that the degree of dispersion is also very important [19].

Numerical simulation has been widely used in concrete. When conducting numerical simulation of concrete, it is usually considered to contain three phases: cement paste, ITZ, and aggregates [20]. Several kinds of numerical models have been established to study the properties of cementitious materials, such as elastic modulus [12], compressive strength [21], tensile strength [22], plastic-damage response [23], and chloride ion transport [24]. Properties of each phase will affect the acquisition of accurate conclusions. Especially the ITZ will have a deep impact on the performance of concrete [25,26]. Furthermore, Wang et al. [27] revealed that NS enhances the mechanical properties of ITZ and mortar, through the numerical simulation, and the concrete added with 2% NS has the better resistance to external force than that without NS.

As for the experimental method related to the cement paste with nanomaterials, compressive, flexural, and tensile properties are often used to verify the macroscopic mechanical properties [28,29,30]. Concrete water absorption [11,31], chloride penetration, and freezing–thawing resistance [15,32] are used to test the durability. Moreover, nanoindentation is mainly applied to test the micromechanical properties [12,33,34].

In this study, the DIC technology was applied to carry out the micromechanical properties of the cement paste with nanomaterials. Different proportions were added to study the effect of NS on the cement paste. By comparing the effects of different NS content, the optimal dosage can be obtained. Finally, the microstructure of the cement paste was also observed using SEM.

## 2. Experimental

### 2.1. Materials

**Figure 1:**
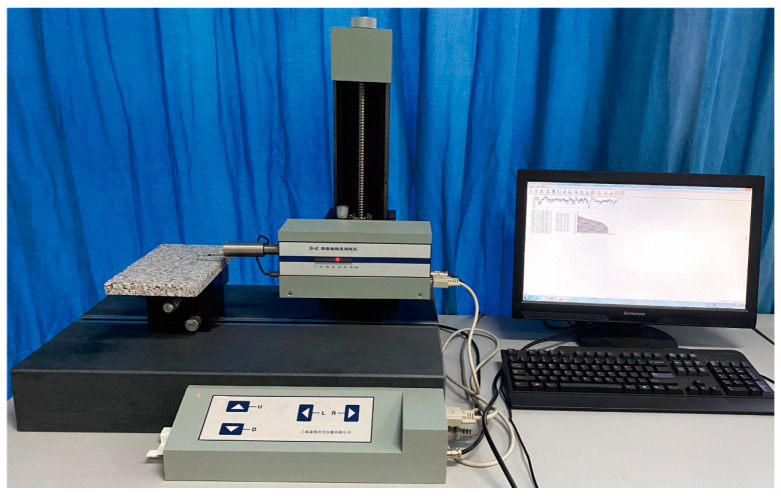
Precision roughness meter.

Ordinary Portland cement (OPC) P·I 42.5 (Fushun Cement Co., Ltd. (Fushun, China)) and NS (Shanghai Yuanjiang Chemical Co., Ltd. (Shanghai, China)) were used as raw materials, their chemical compositions, and their physical properties are listed in Table 1, Table 2 and Table 3, respectively. Granite slabs were used as aggregate, and their surface maintained the natural roughness after cutting. Aggregate surface roughness has a significant effect on the ITZ [35]. Therefore, the granite used for the specimen was kept the same, so that the influence caused by roughness could be minimized. Surface roughness is determined by the parameter *R*_a_, which represents the arithmetical mean deviation of surface profile [36]. The *R*_a_ value of granite was measured using a precision roughness meter, as shown in Figure 1. The test result was 2.931 ± 0.359 μm (mean ± SD).

The mixed proportion of the specimens is shown in Table 4. For all groups, the water/binder (*w*/*b*) ratio was maintained at 0.5, and the superplasticizer (Sobute New Materials Co., Ltd. (Nanjing, China)) dosage was maintained at 0.5% by weight of cement. The proportion of nanomaterials was considered as the only variable among each group.

It should be noted that, in the DIC test, cement paste was used instead of concrete as the base material, which can reduce the impact of other factors. Nevertheless, in the SEM test, to better observe the microstructure of the ITZ, the mortar sample was prepared in accordance with GB/T 17671-2021 [37]. Standard sand was used for the sample preparation, and the mix proportion of other components remained unchanged.

### 2.2. Specimen Preparation

The specimen preparation in this paper was consistent with that of reference [38]. Broadly speaking, the cement paste with different mix proportions was poured onto the granite slab. After curing for 28 d, samples were cut into small specimens for the DIC test. The main difference lay in the treatment of NS. First of all, NS and superplasticizer were added to water, and then sonicated for 15 min. The addition of superplasticizer not only contributed to the dispersion of flocculation structure, but also mainly reduced the adsorption of water molecules of cement particle surface [39]. Before casting, they were added to the cement for further mixing with an electric mixer.

After standard curing for 28 d, specimens were cut to a size suitable for the axial compression test. Ultimately, six specimens per group were required for the loading test, half of them were used for the cement paste properties test and half for the ITZ test. The method of making artificial speckles was the same as in previous research.

### 2.3. Experimental Procedure

#### 2.3.1. Flowability

The mini-slump cone test, according to GB/T8077-2012 [40], was carried out to assess the flowability of the cement paste. The prepared cement paste was loaded into the standard truncated cone mold, then the mold was vertically lifted, and the maximum diameters of two orthogonal directions of the flow spread were measured 30 s later. The average value was taken as the flowability of the cement paste.

#### 2.3.2. Axial Compression Test

**Figure 2:**
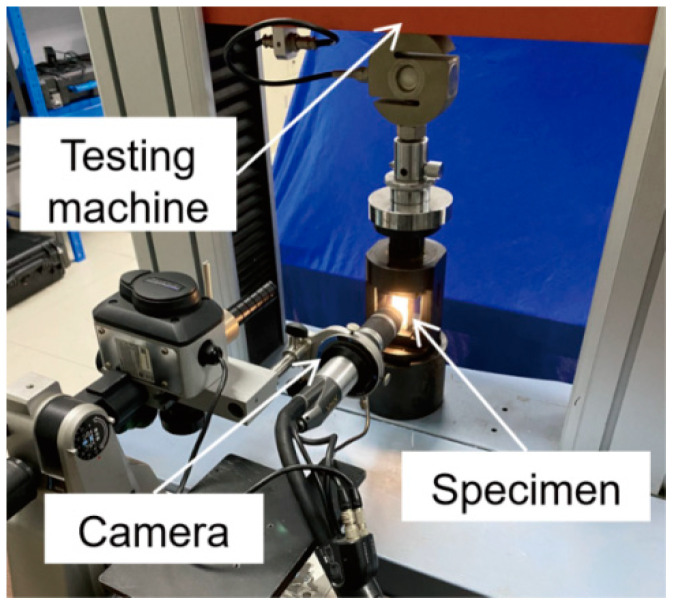
The experimental setup.

Based on the reference [38], the axial compression test was carried out by a universal testing machine (20 kN), and the loading rate was 0.05 mm/min. According to the bearing capacity test, the final load was also 10,000 N. During the compression test, the CCD camera was applied to collect digital images of the corresponding observation area, as illustrated in Figure 2.

**Figure 3:**
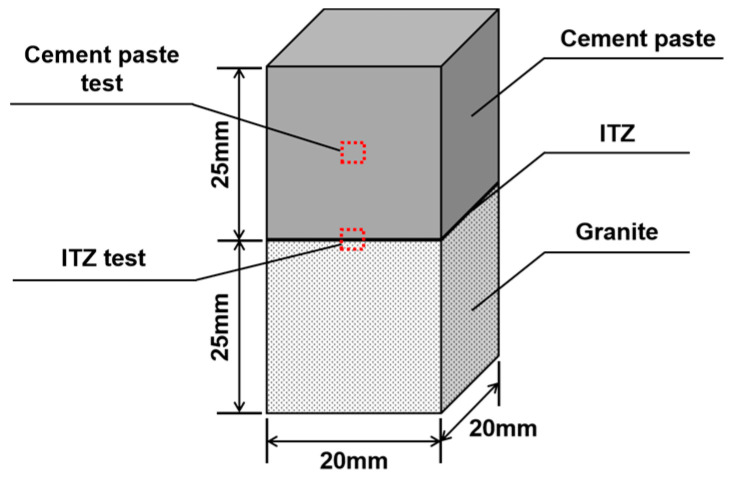
Geometry and observation areas of the specimen.

For specimens used to test the cement properties, the observation area was placed in the central area of the cement paste, as shown in Figure 3. Similarly, for the ITZ, the observation area was at the center of the interface. Each specimen was loaded only once.

#### 2.3.3. Basics of DIC

By calculating the correlation of digital images observed before and after deformation, the corresponding displacement and strain fields are obtained [41]. Specifically, a subset was determined in the reference digital image before deformation. Then, the mathematical function, called the cross-correlation coefficient, was used to find the most similar image subset on the deformed image. In this paper, the zero-mean normalized cross-correlation function can be expressed as follows:

(1) $$C=\frac{\sum \sum \left[f\left(x,y\right)-\bar{f}\right]\cdot \left[g\left(x^{\prime },y^{\prime }\right)-\bar{g}\right]}{\sqrt{{\sum \sum \left[f\left(x,y\right)-\bar{f}\right]}^{2}\cdot {\sum \sum \left[g\left(x^{\prime },y^{\prime }\right)-\bar{g}\right]}^{2}}}$$

where *f*(*x*, *y*) is the gray value at coordinate (*x*, *y*) for the reference image; *g*(*x*′, *y*′) has an equivalent definition but for the deformed image; and $\bar{f}$ and $\bar{g}$ are the average gray values of the reference and deformed images, respectively.

After the target subset was matched, the displacement function of the subset is most commonly expressed as follows:

(2) $$\left\{\begin{array}{l} x^{\prime }=x+u+\frac{\partial u}{\partial x}\Delta x+\frac{\partial u}{\partial y}\Delta y\\ y^{\prime }=y+v+\frac{\partial v}{\partial x}\Delta x+\frac{\partial v}{\partial y}\Delta y \end{array}\right.$$

where *u* and *v* are the *x*- and *y*-directional displacement components of the reference sub-region center towards the deformed position. Also, Δ*x* and Δ*y* are the distances from the point (*x*′, *y*′) to the center of the image sub-region (*x*, *y*). Meanwhile, *∂u*/*∂x*, *∂u*/*∂y*, *∂v*/*∂x*, and *∂v*/*∂y* are the first derivatives of the displacements.

It is noteworthy that by smoothing the computed displacement field first and then calculating the strain, the accuracy of strain estimation will be improved [42]. The local least-squares fitting technology for strain estimation was used to compute the strain. With the application of the Vic-2D v6 software (Correlated Solutions, Inc., Irmo, SC, USA), the displacement and strain field can be calculated. 

#### 2.3.4. SEM

Field emission scanning electron microscopy (Zeiss Gemini SEM 300) (Carl Zeiss Ltd., Cambridge, UK) was used to characterize the microstructure of hardened cement paste, which would further confirm the conclusions of mechanical experiments.

## 3. Results and Discussion

### 3.1. Influence of NS Content on the Flowability

**Figure 4:**
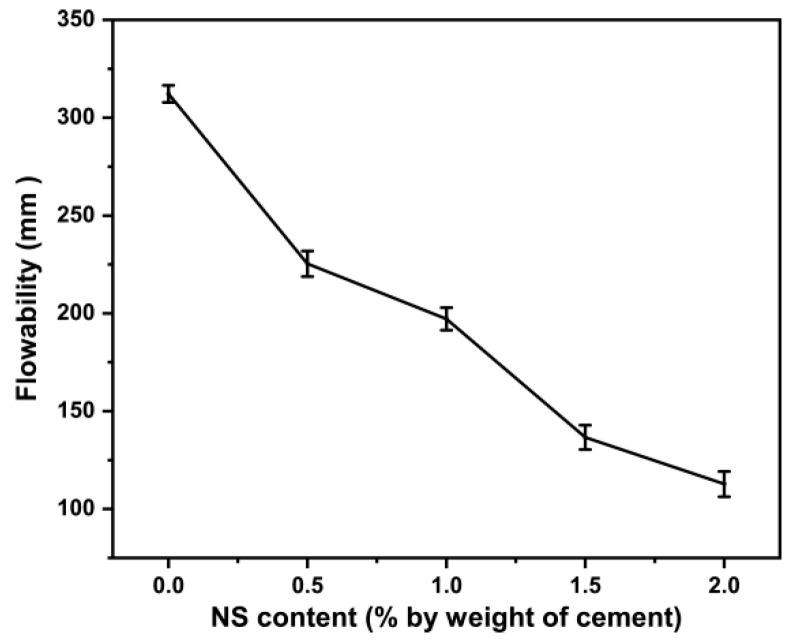
Influence of NS content on flowability.

Water is the fundamental factor affecting the flowability of the cement paste. Water first filled the packing voids of cement particles and then formed the water coating film. As the amount of water increased, the excess water would increase the paste flowability [39]. Figure 4 presented the change of flowability of the cement paste with NS. With the increase in NS content, the flowability decreased continuously. Specifically, when the content is 0.5% and 1.5%, respectively, the flowability of cement paste was significantly reduced compared with the previous state. Comparing the flowability of the NS0 and NS2, the value decreased from 312.2 mm to 112.8 mm. As mentioned in references [43,44,45], the nanoparticles formed loose flocculated and coated layers around cement particles, which absorbed some free water that originally contributed to flowability. It is worth noting that the incorporation of superplasticizer may affect the flowability of the paste. A classical explanation can be that the added superplasticizer was adsorbed on the surface of cement and nanomaterial particles, yielding a negative surface charge. The electrostatic repulsion hindered aggregation and decreased the flocculation effect, and the water inside the agglomeration was released [39,46].

### 3.2. DIC Analysis

**Figure 5:**
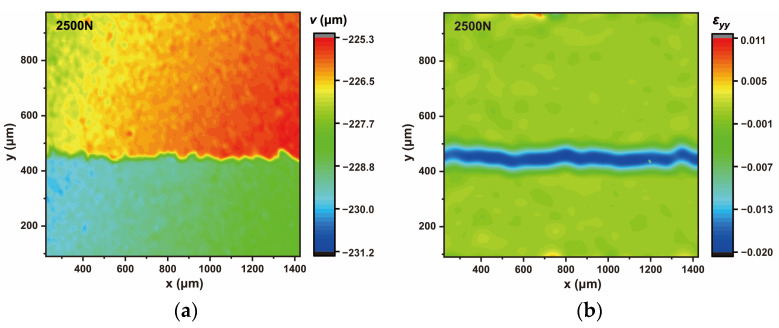
DIC results for NS0.5 under the load of 2500 N. (**a**) Displacement field (*v*) in the *y*-direction. (**b**) Strain field (*ε_yy_*) in the *y*-direction.

**Figure 6:**
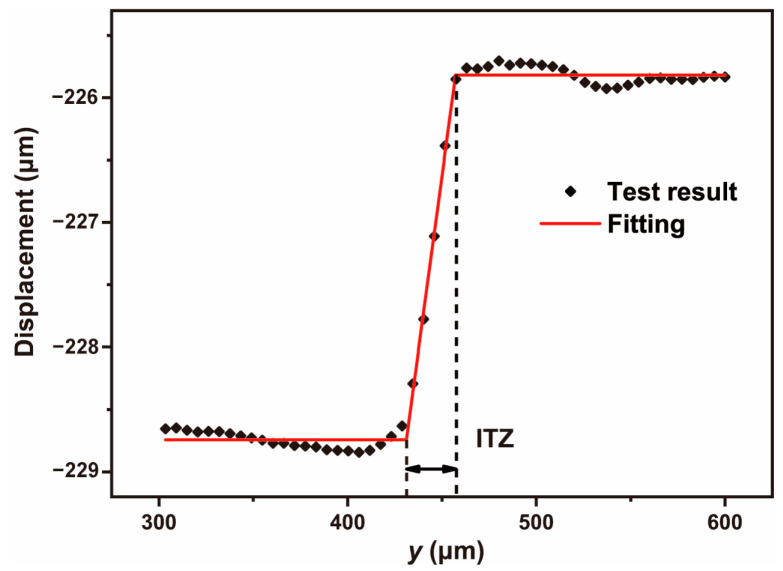
Piecewise fitting of displacement curve.

The vertical displacement (*v*) and strain (*ε_yy_*) values in the observation area were obtained with DIC technology. Typical vertical displacement (*v*) and strain (*ε_yy_*) field of NS0.5 were shown in Figure 5. In the vertical displacement field, because of the difference in mechanical properties of each phase, their displacement changes were different as well. Same as in reference [38], the displacement data were extracted and fitted by the piecewise function. As shown in Figure 6, the jumping area between two horizontal lines represents the ITZ. The *y*-direction between the two inflection points is the thickness of the ITZ. In the strain field, strain values under different compressive loads were extracted from the DIC analysis results. By fitting the curve during the elastic stage, the corresponding elastic modulus can be calculated. This method was also applicable to cement paste and the ITZ.

It should be pointed out that the DIC experimental results showed a high dispersion. Therefore, it was necessary to use statistical methods for further analysis, and make sure that the sample size of each group was not less than 30. The high dispersion may be caused by the following reasons: (1) cement paste and granite are heterogeneous materials and (2) the experiment was carried out on the mesoscopic level; hence, small changes could also lead to a high dispersion.

#### 3.2.1. Elastic Modulus of the Cement Paste with NS

**Figure 7:**
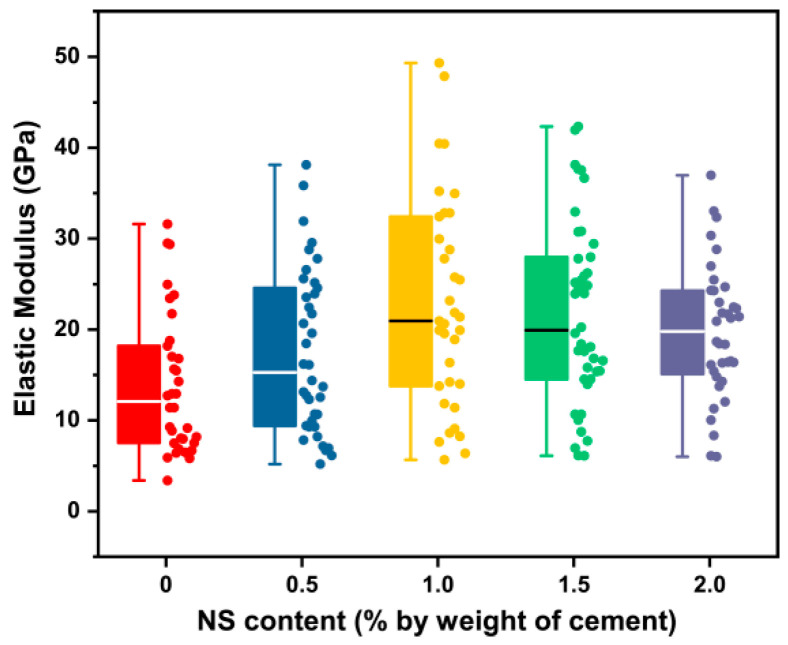
Influence of NS content on the elastic modulus of the cement paste.

As can be seen in Figure 7 and Table 5, the elastic modulus of the cement paste increased with the gradual increase in NS content. According to the Shapiro–Wilk test, the data sample did not follow the normal distribution, so the median was used as the result of each group. Groups NS0.5, NS1, NS1.5, and NS2 showed an increase of 26.42%, 73.50%, 65.05%, and 63.86%, respectively, when compared to NS0. The maximum value among them was 20.95 GPa from Group NS1. The numerical range of the elastic modulus obtained using the DIC experiment was consistent with references [47,48]. Statistical analysis implied that there were significant differences between Group NS0 and other groups (*p* < 0.05), except for NS0.5 (*p* = 0.916 > 0.05). This result confirmed that after the addition of NS, the enhanced effect was gradually produced, and the best effect was achieved at 1%. It was attributed to the filling effect and the high pozzolanic reactivity of NS to improve the strength [49]. When the NS content exceeded 1%, the elastic modulus of the cement paste slightly decreased. This may be related to the agglomeration that was difficult to disperse, even with the addition of superplasticizer and ultrasonic treatment. It should be pointed out that superplasticizer will have an impact on the mixing of cement slurry, the dispersion of nanomaterials, and even the mechanical properties of cement paste. Therefore, we took the content of NS as the only variable and kept the content of superplasticizer as a constant, so as to reduce the additional effect of superplasticizer.

#### 3.2.2. Elastic Modulus of the ITZ with NS

**Figure 8:**
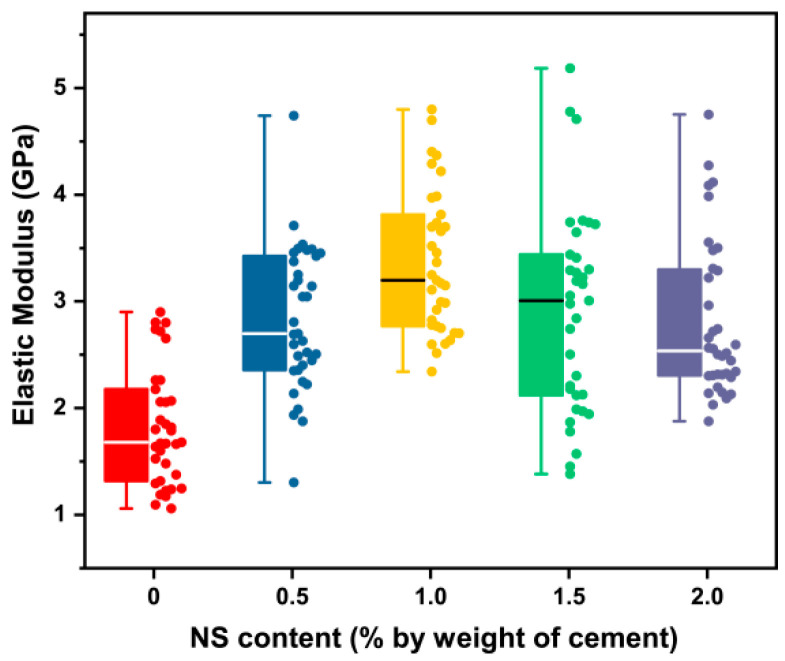
Influence of NS content on the elastic modulus of the ITZ.

Figure 8 and Table 6 summarized the statistical analysis results of NS content on the elastic modulus of the ITZ. As the NS content increased, the elastic modulus of the ITZ increased first, and then showed a decreasing trend when the NS content exceeded 1%. It was obvious that NS has a significant effect on the mechanical properties of the ITZ, since the *p*-values between Group NS0 and the other four groups were less than 0.05. Moreover, there was a significant difference between Group NS1 and NS2 (*p* < 0.05), which implied that excess nanomaterials would have significant adverse effects. To be specific, Group NS0.5, NS1, NS1.5, and NS2 increased by 60.72%, 90.50%, 78.95%, and 51.22%, respectively, when compared to NS0. The maximum elastic modulus was 3.20 GPa, which was derived from group NS1. The reasons for this phenomenon were as follows: (1) The smaller particle size of the NS filled the pores between the cement particles. (2) NS can consume excess CH, promote the hydration of cement, and then improve the mechanical properties of the paste [26]. (3) When the NS content is larger than 1%, the agglomeration of nanomaterials could become significant and have a negative effect. Thus, for the ITZ, the optimal addition range is about 1%.

Compared with the previous section, the increased amplitude of the elastic modulus of the ITZ was larger than that of the cement paste, except for Group NS2. This is because there are many more pores and defects in the ITZ, so NS can provide more remarkable improvements. Similar trends were also observed by Deependra et al. [2].

#### 3.2.3. Thickness of the ITZ with NS

**Figure 9:**
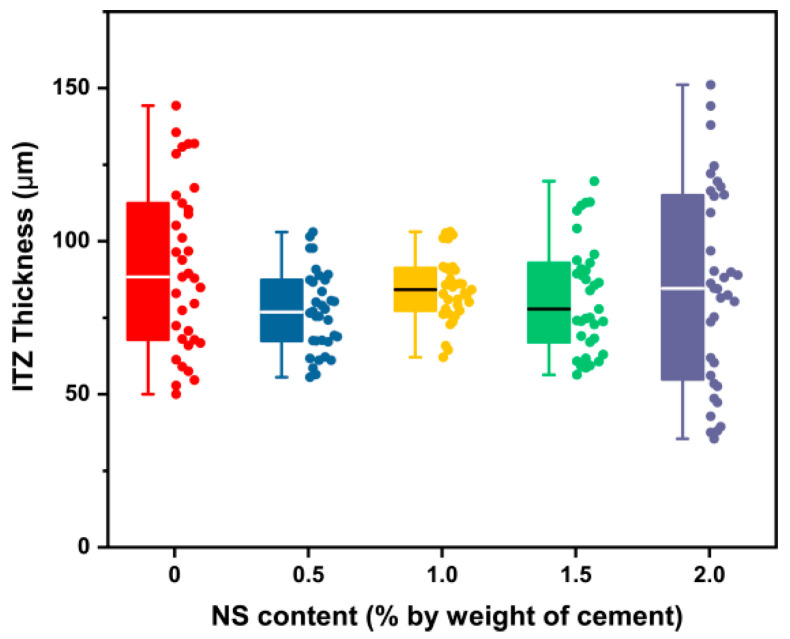
Influence of NS content on the thickness of the ITZ.

The influence of NS on the thickness of ITZ is presented in Figure 9 and Table 7. Statistical analysis showed that differences between each group were not significant. As explained in the previous sections, NS would certainly promote the mechanical properties of the ITZ. Even so, the elastic modulus of the ITZ remained smaller than that of the cement paste. Therefore, the boundary of each phase can still be distinguished by the change of displacement. Xu et al. [50] also confirmed the conclusion that the influence of NS on the interface width was not significant, and its impact on nanomechanical properties of the interface was marked.

As determined with the Shapiro–Wilk test, the results of each group followed a normal distribution; thus, the mean value was considered as the ITZ thickness. And the thickness value was in the range of 76.91–91.38 μm.

### 3.3. SEM Analysis

**Figure 10:**
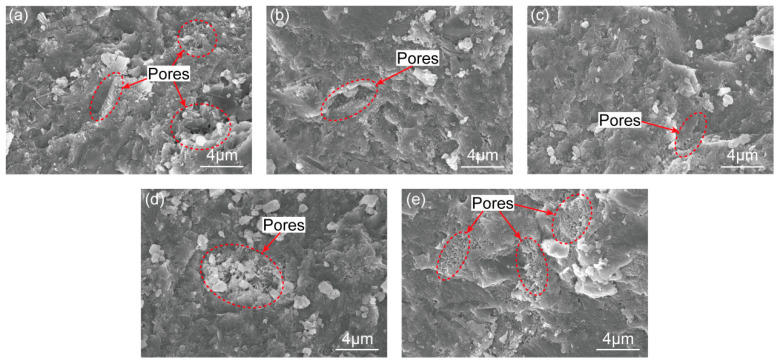
SEM images of the cement paste: (**a**) NS0; (**b**) NS0.5; (**c**) NS1; (**d**) NS1.5; and (**e**) NS2.

SEM analysis is beneficial to study the cause of the mechanical characteristics’ change of the cement paste and the ITZ. As illustrated in Figure 10a–e, when NS content changed from 0–1.0%, the microstructure of the paste became denser. It is well known that NS particles can not only be used as void fillers to improve the microstructure, but also to promote the pozzolanic reaction [2,9,51]. However, when the NS dosage was larger than 1%, some pores filled with needle-hydrates can be observed. This phenomenon can be explained by the agglomeration of excess nanomaterials, limiting the formation of uniform hydrate microstructure and leading to low strength [52]. The decrease in the elastic modulus obtained in the previous sections has a good relationship with the SEM study.

**Figure 11:**
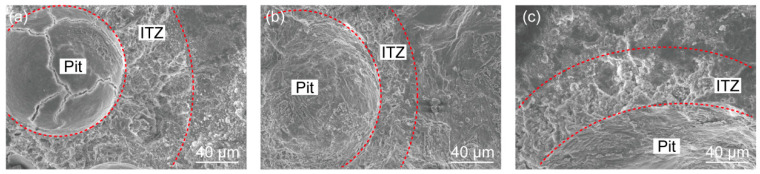
SEM images of the ITZ: (**a**) NS0; (**b**) NS1; and (**c**) NS2.

To study the microstructure of ITZ, the broken pieces of the mortar sample were selected for SEM. Since the diameter of sand particles is much larger than that of cement and nanomaterial particles, ITZ can also be generated on the surface of sand particles. As revealed in Figure 11, we mainly focused on the edge of the small pit formed after the sand particles were removed. From this perspective, both the porous structure of the ITZ and the microstructure of the interface between the cement paste and sand particle can be observed.

Compared with the cement paste, the porous structure around the sand grains was more obvious, as can be seen from Figure 11. The cement paste gradually became dense with an increase in the distance from the sand particle’s surface [53,54]. The interior of the sand particle pit was dense and smooth on the whole. This is mainly due to the “wall effect”, and the local water is sufficient for the hydration reaction so that the sand grains can be well wrapped by cement paste [55]. Yet there were some cracks, pores, and unhydrated cement particles, which is inevitable. Moreover, some wide cracks in the sand particle pit might be caused by the self-shrinking of the cement paste. Figure 11a–c displayed the typical ITZ microstructure at the NS content of 0, 1%, and 2%, respectively. The ITZ became denser, after the NS was added in. However, because of the complex porous structure, it is difficult to quantify the thickness of ITZ using SEM.

## 4. Conclusions

In this paper, the influence of NS content on the cement paste and ITZ was investigated with the DIC technology. The main conclusions can be summarized below:

1. The flowability of the cement paste decreased continuously, as the NS content increased, under the condition of superplasticizer and ultrasonic treatment. Comparing the flowability of the NS0 and NS2, the value decreased from 312.2 mm to 112.8 mm. In the content range of 0.5–1%, the flowability showed good working performance.
2. NS can effectively improve the mechanical properties of cement paste. When the NS content was 1%, the elastic modulus increased the most, about 73.50%, compared with Group NS0. The maximum value among them was 20.95 GPa from group NS1. When the content exceeded 1%, the elastic modulus slightly decreased.
3. For the ITZ, NS can greatly promote its mechanical properties. When 1.0% NS was incorporated, its elastic modulus increased by 90.50%. The maximum value is 3.20 GPa, also from NS1. However, NS content has no significant effect on the thickness of the ITZ, and the thickness value was in the range of 76.91–91.38 μm.
4. SEM confirmed that NS densified the microstructure of the cement paste and the ITZ. Compared with the cement paste, the porous structure around the sand grains was more obvious. The cement paste gradually became dense with the increase in the distance from the sand particle’s surface.

To sum up, when applying NS to enhance the mechanical properties of the cement paste, 1% can be used as the optimal content. Of course, treatments conducive to the dispersion of NS are also necessary. In this paper, the results about the cement paste and ITZ can provide reference for relevant numerical simulations. Likewise, based on the DIC technology, effects of various nanomaterials on cementitious materials can be deeply studied. This work will provide the basis for the application of nanomaterials in the field of engineering.

## Figures and Tables

**Table 1:** Chemical composition of OPC (%).

Oxides						Other Components	Ignition Loss
SiO_2_ 20.96	Al_2_O_3_ 4.13	Fe_2_O_3_ 3.03	CaO 62.32	MgO 2.90	SO_3_ 2.38	1.436	2.14

**Table 2:** Physical properties of OPC.

Specific Surface Area (m^2^/kg)	Density (kg/m^3^)	Standard Consistency (%)	Setting Time (min)		Flexural Strength (MPa)	Compressive Strength (MPa)
358	3110	26.00	Initial	Final	3 days 5.9	3 days 28.0
			107	167		

**Table 3:** Physical properties of NS.

Diameter (nm)	Surface-to-Volume Ratio (m^2^/g)	Density (g/cm^3^)	Purity (%)
50	210	2.41	99.9

**Table 4:** Mix proportion of the specimens (kg/m^3^).

No.	*w*/*b*	Mix Proportion of the Specimens			
		Water (g)	Cement (g)	NS (g)	Superplasticizer (% by Weight of Cement)
NS0	0.5	225	450	-	0.5
NS0.5	0.5	225	447.75	2.25	0.5
NS1	0.5	225	445.5	4.5	0.5
NS1.5	0.5	225	443.25	6.75	0.5
NS2	0.5	225	441	9	0.5

**Table 5:** Statistical results for the elastic modulus of the cement paste.

NS Content (% by Weight of Cement)	0	0.5	1.0	1.5	2.0
Mean	13.82	17.45	22.79	21.80	19.76
Standard deviation	7.70	9.01	11.70	10.11	7.40
Minimum	3.38	5.18	5.67	6.09	5.99
Median	12.08	15.27	20.95	19.93	19.79
Maximum	31.61	38.13	49.33	42.32	36.99
Interquartile range (Q3–Q1)	10.82	15.33	18.64	13.82	9.34
Range (Maximum–Minimum)	28.23	32.95	43.66	36.23	30.99

**Table 6:** Statistical results for the elastic modulus of the ITZ.

NS Content (% by Weight of Cement)	0	0.5	1.0	1.5	2.0
Mean	1.82	2.83	3.36	2.90	2.81
Standard deviation	0.55	0.67	0.67	0.95	0.73
Minimum	1.06	1.30	2.34	1.38	1.88
Median	1.68	2.70	3.20	3.01	2.54
Maximum	2.90	4.74	4.80	5.18	4.75
Interquartile range (Q3–Q1)	0.86	1.07	1.05	1.32	1.00
Range (Maximum–Minimum)	1.84	3.44	2.46	3.80	2.88

**Table 7:** Statistical results for the thickness of the ITZ.

NS Content (% by Weight of Cement)	0	0.5	1.0	1.5	2.0
Mean	91.38	76.91	84.25	81.80	84.71
Standard deviation	27.07	13.13	10.44	18.09	32.65
Minimum	49.98	55.55	62.11	56.39	35.45
Median	88.36	76.83	84.21	77.84	84.57
Maximum	144.34	102.98	103.14	119.63	151.16
Interquartile range (Q3–Q1)	44.63	19.94	13.91	25.92	60.93
Range (Maximum–Minimum)	94.36	47.43	41.03	63.24	115.71

## Data Availability

Not applicable.
